# Intensified East Asian winter monsoon during the last geomagnetic reversal transition

**DOI:** 10.1038/s41598-019-45466-8

**Published:** 2019-06-28

**Authors:** Yusuke Ueno, Masayuki Hyodo, Tianshui Yang, Shigehiro Katoh

**Affiliations:** 1 Department of Planetology, Kobe University, Kobe, 657-8501 Japan; 2 Research Center for Inland Seas, Kobe University, Kobe, 657-8501 Japan; 3 China University of Geosciences, Beijing, China; 4 Museum of Nature and Human Activities, Hyogo, Sanda, 669-1546 Japan

**Keywords:** Climate change, Palaeoclimate

## Abstract

The strength of Earth’s magnetic dipole field controls galactic cosmic ray (GCR) flux, and GCR-induced cloud formation can affect climate. Here, we provide the first evidence of the GCR-induced cloud effect on the East-Asian monsoon during the last geomagnetic reversal transition. Bicentennial-resolution monsoon records from the Chinese Loess Plateau revealed that the summer monsoon (SM) was affected by millennial-scale climate events that occurred before and after the reversal, and that the winter monsoon (WM) intensified independently of SM variations; dust accumulation rates increased, coinciding with a cooling event in Osaka Bay. The WM intensification event lasted about 5000 years across an SM peak, during which the Earth’s magnetic dipole field weakened to <25% of its present strength and the GCR flux increased by more than 50%. Thus, the WM intensification likely resulted from the increased land–ocean temperature gradient originating with the strong Siberian High that resulted from the umbrella effect of increased low-cloud cover through an increase in GCR flux.

## Introduction

Records of suborbital-scale climate variation during the last glacial and Holocene periods can be used to elucidate the mechanisms of rapid climate changes^1,2^. Centennial- to decadal-resolution paleoceanic records^3–6^ recently revealed multiple suborbital-scale climate events in the marine isotope stage (MIS) 19 interglacial period, when the last geomagnetic reversal occurred. Most events were associated with sea-level changes, some possibly related to iceberg discharge^5^. At least one event was associated with a decrease in the strength of the Earth’s magnetic field^6^. Thus, climate records from the MIS 19 interglacial can be used to elucidate the mechanisms of a variety of climate changes, including testing the effect of changes in geomagnetic dipole field strength on climate through galactic cosmic ray (GCR)-induced cloud formation^6–8^, as the present evidence for this effect is weak and biased toward the oceans.

**Figure 1:**
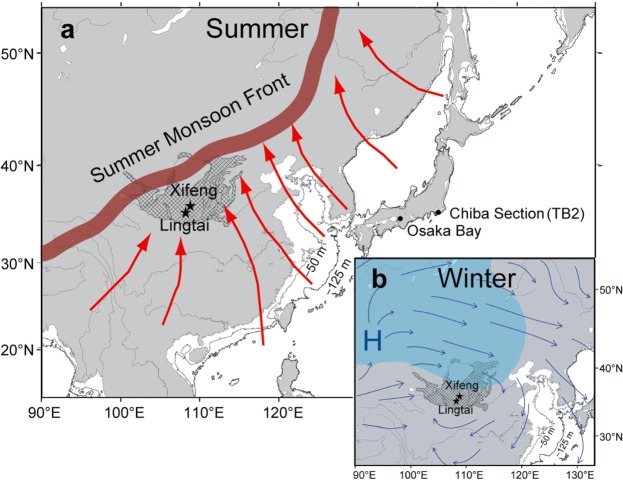
Map of the East-Asian monsoon area. The hatched area shows the Chinese Loess Plateau. The stars indicate Lingtai (35.04°N, 107.39°E) and Xifeng (35.45°N, 107.49°E). The maps are drawn using the GMT version 4 (http://www.soest.hawaii.edu/gmt/). The coastlines for sea levels at −125- and −50-m elevations are shown by the thick and thin lines, respectively. (**a)** The summer monsoon front (brown belt) and summer monsoon directions (red arrows) are after Porter and An^13^. (**b)** The Siberian High (blue area) and the winter monsoon directions (blue arrows) are after Hao *et al*.^23^.

The East-Asian monsoon consists of the summer monsoon (SM), characterized by the influx of warm, moist wind from the Pacific air mass, and the winter monsoon (WM), characterized by the influx of cold, dry wind from the Siberian air mass (Fig. 1). The SM strengthens and the WM weakens during warm, moist interglacial periods, and the opposite occurs during cold, dry glacial periods^9–11^. Past East-Asian monsoon variations are recorded in loess-paleosol sequences on the Chinese Loess Plateau (CLP), where aeolian dust transported from deserts mainly north–northwest by the WM has been deposited since about 2.6 Ma^12^. High summer precipitation causes pedogenesis of loess during interglacial periods, whereas pedogenesis is weaker during glacial periods because of low summer precipitation. Therefore, the loess and paleosol layers correspond to glacial and interglacial periods, respectively^9^. In addition to the orbital-scale responses, the WM responded to millennial-scale cold events during the last glacial period^13,14^.

In this study, we examined the East-Asian monsoon response to millennial-scale climate events during the MIS 19 interglacial using high-resolution magnetic susceptibility (χ), the frequency dependence of magnetic susceptibility (χ_FD_), and grain size records from Xifeng and Lingtai in the CLP (Fig. 1) as proxies of monsoon intensity. High-resolution Matuyama–Brunhes (MB) transition records were previously obtained from the same sections of these areas^15,16^. Both records contain a multiple polarity flip (MBpf) zone, the climatostratigraphic position of which is consistent with many other regions in the CLP^17,18^, and which may correlate to similar zones in marine and lacustrine sediments^19–21^.

## Results

### Vertical changes of monsoon proxies

**Figure 2:**
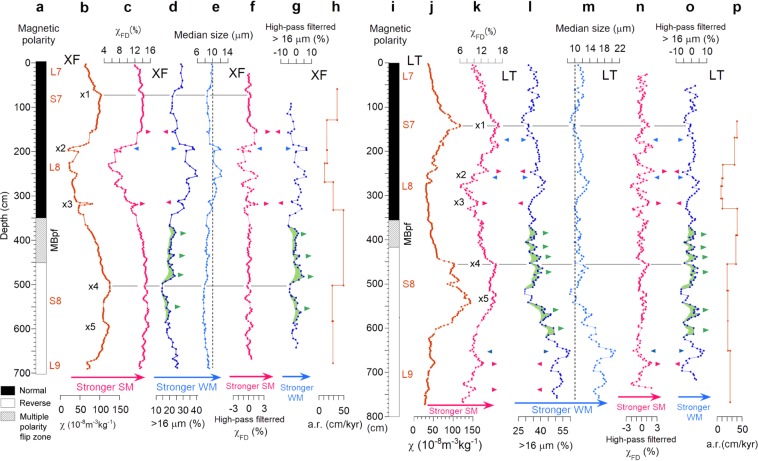
Vertical plots of monsoon proxies and accumulation rates. (**a**‒**h**) Xifeng; (**i**‒**p**), Lingtai. (**a**,**i**), Magnetic polarity. MBpf, Matuyama–Brunhes polarity flip zone. (**b**,**j**) Magnetic susceptibility (χ). (**c**,**k**) Frequency dependence of magnetic susceptibility (χ_FD_). (**d**,**l**) Coarse grain content (CGC). (**b**,**e**) Median grain size. (**f**,**n**) High-pass-filtered χ_FD_. (**g**,**o**) High-pass-filtered CGC. (**h**,**p**) Accumulation rates between age control points. Paired arrowheads indicate summer (SM) and winter (WM) monsoon inverse correlations. WM maxima without correlation with SM variations are in green. “x1” to “x5” are notable features (see main text).

The χ and χ_FD_ of the SM proxies in Xifeng were 20–130 × 10^−8^ m^3^ kg^−1^ and 5–16%, respectively (Fig. 2b,c). The coarse (>16 μm) fraction and median grain size of the WM proxies in Xifeng were 14–40% and 7–12 μm, respectively, showing an inverse correlation with SM in the long wavelengths of the strength changes (Fig. 2d,e; Supplementary Fig. S1). Inverse correlation was also observed in short episodes (<30 cm), as indicated by arrowheads in Fig. 2c,d, and is more clearly visible in the high-pass filtered proxies (Fig. 2f,g). The SM and WM changed rapidly with the inverse correlation, particularly in episodes “x2” at 190–200 cm and “x3” at 315–320 cm. In contrast, the WM changed at a high frequency without correlation with the SM between 370 and 580 cm, with several maxima (green in Fig. 2d,g). This interval lies across the SM peak, partially overlapping the MBpf zone (Fig. 2a).

The χ and χ_FD_ in Lingtai were similar to those in Xifeng (Fig. 2j,k), whereas the average grain size was coarser, due mainly to the lowermost coarse portion below about 600 cm (Fig. 2l,m). The SM and WM in Lingtai also showed inverse correlations in the short episodes between 170 and 320 cm and below 650 cm (Fig. 2k–o). However, there were no inverse correlations in the long-wavelength changes between 340 and 540 cm in Lingtai (Supplementary Fig. S1). In and below the MBpf zone, the WM showed oscillatory intensification with no correlation with the SM, as highlighted in green for the maxima in Fig. 2l,o.

The SM proxies of χ and χ_FD_ showed consistent variation, with both having a sharp SM peak in the S7 layer and double SM peaks in the S8 layer, designated “x1”, “x4”, and “x5”, respectively, and a short SM minimum of L8 at 228 cm in Xifeng and 281 cm in Lingtai (Fig. 2b,k). The MBpf zone is between “x3” and “x4”. The short episodes with anti-phase SM and WM variations are concentrated around L8, where there are some episodes without an anti-phase companion, suggesting that the episodes have multiple causes.

### Precession-cycle monsoon changes

Changes in χ in the Chinese loess-paleosol sequences are consistent with marine oxygen isotope ratios, which are proxies of land ice volume and glacial sea-level changes^9^. In addition to effects on global climate linked to ice volume changes, the movement of the SM front between interglacial and glacial periods in response to the regression of coastlines is a possible influential mechanism (Fig. 1)^22^. Thus, the orbital-scale SM maxima correlated with the peaks of glacial sea-level changes^10,23^. Considering the timing of the MB reversal in sea-level changes^24^, the SM peaks of S8 and S7 and the SM minimum of L8 correlate with the precession-cycle highstand MISs 19.3 and 19.1 and lowstand MIS 19.2, respectively. These correlations are different from most of the previous studies^23,25^ that correlated paleosol layer S7 with MIS 19, and layer S8 with MIS 21. That required the assumption of unlikely, large lock-in depths for magnetization acquisition to interpret the large downward shift of the MB boundary to the previous interglacial layer interposing the glacial layer. Our correlation scheme is based on the small lock-in depth supported by laboratory experiments and field observations (see Method for details)^26–29^.

**Figure 3:**
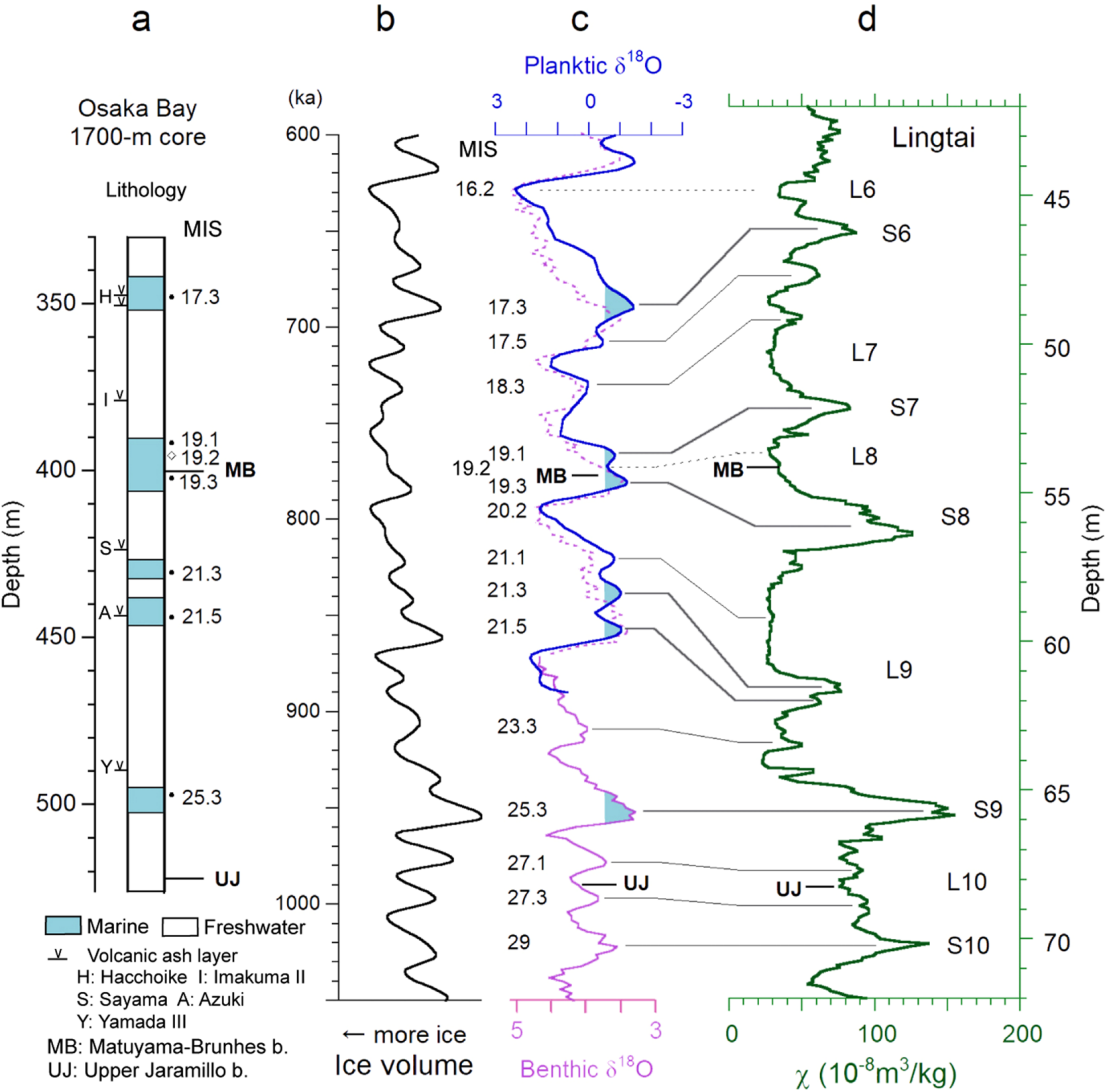
Correlation of precession-cycle sea-level changes. (**a**) Marine layers in the 1700-m-long Osaka Bay core^4,6,40^. The dots show highstand and the open diamonds represent lowstand. (**b**) Ice volume model calculated following Lisiecki and Raymo^31^. (**c**) Planktic δ^18^O of the low latitude stack^33^ (blue line) and benthic δ^18^O of stack LR04^31^ (purple line). The planktic δ^18^O curve is tuned to the ice volume model in (**b**). The light-blue areas show the estimated intervals of sea level exceeding the Osaka Bay sill (−48 ± 4 m elevation). (**d**) Magnetic susceptibility from Lingtai^25^.

Paleosol layer S7 is often correlated with a highstand in MIS 18, and layer S8 with MIS 19^18,30^. Such correlations may stem from the faint signals of MISs 19.2 and 19.1 in benthic marine oxygen isotope data^21,31^; however, these signals are clear in high-resolution marine oxygen isotope data^3,5^. The high-resolution diatom-based sea-level variation records from Osaka Bay also show these signals, and definitively constrain the precession-cycle sea-level changes (Fig. 3)^4,6,24^. The sea levels during highstand MISs 19.1, 19.3, and 17.3 and lowstand MIS 19.2 clearly exceeded the Osaka Bay sill (−48 ± 4 m elevation)^4^. In contrast, those of highstand MISs 18.3, 18.1, 17.5, and 17.1 were evidently below the sill because the corresponding marine layers are absent in the sequence, despite the lack of a hiatus in deposition^32^. These sea-level constraints are consistent with the planktonic marine oxygen isotope variations in the low-latitude stack (Fig. 3)^33^, and even post–MIS 17 precession-cycle sea-level changes correlate well with the isotope variations in the stack^34,35^.

### Millennial-scale monsoon changes

**Figure 4:**
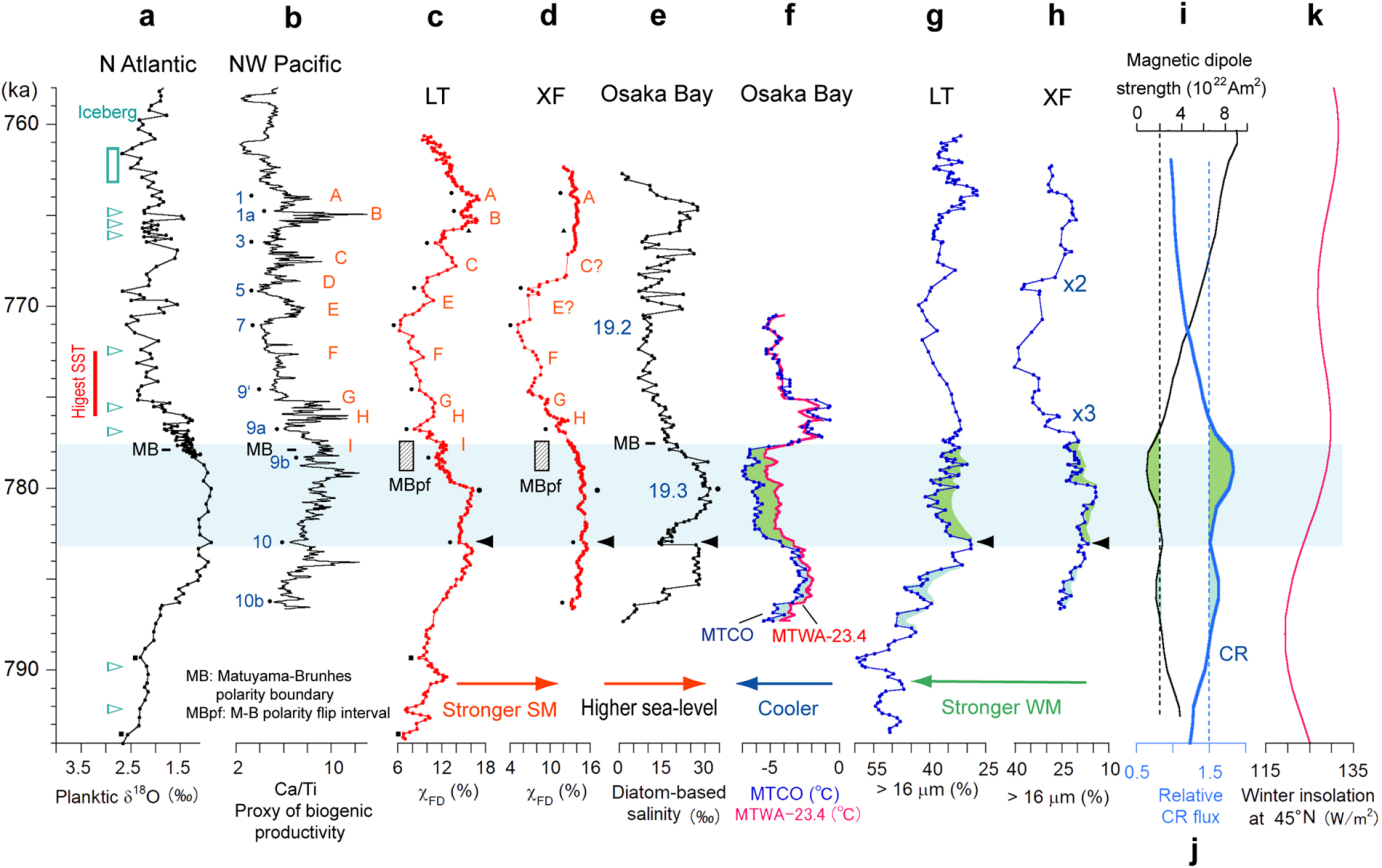
Comparison with other paleoclimate proxies. (**a**) Planktonic δ^18^O^3^ plotted to the age model^5^. (**b**) Bioproductivity proxy^5^. The labels follow Hyodo *et al*.^5^, except 1a and 9′, defined in this study. (**c**,**d**) Summer monsoon proxies (this study). (**e**) Sea-level proxy^4^. (**f**) Mean temperatures of the warmest (MTWA) (subtracted by 23.4 °C) and coldest (MTCO) months^37^. (**g**,**h**) Winter monsoon proxies (this study). (**i**) Magnetic dipole strength^38^. (**j**) Galactic cosmic ray flux. (**k**) Winter insolation (Dec. 21 to Jan. 21) at 45°N^52^. LT; Lingtai. XF; Xifeng. MB; main Matuyama‒Brunhes boundary. MBpf; Matuyama‒Brunhes polarity flip interval. See the Method for the correlation points (small solid circles, squares, and triangles) and the main text for the colored areas.

The pre-reversal SM double peaks (“x4” and “x5”) correlate well with the MIS 19.3 sea-level highstand and that before the brief sea-level fall, respectively (Figs 2, 4c–e)^4^. Successive post-reversal short-wavelength SM changes are comparable with the millennial- to centennial-scale warming/cooling events associated with sea-level changes (Fig. 4a,b,e; see the Method)^5^. The SM proxy curves show strengthening episodes correlated with most of the short high sea-level/warming events A–I (G and H are warming events without significant sea-level rise) (Fig. 4a–e)^5^. These strengthening episodes lasted for 300‒2200 years and are synchronous between Lingtai and Xifeng, with the different shapes of variation in some episodes reflecting regional differences in sensitivity to SM because of geographic and/or climatic conditions.

These results suggest that the brief high sea-level/warming (low sea-level/cooling) events in the North Atlantic and Northwest Pacific mid-latitudes left traces in the climate in the CLP as SM strengthening (weakening) events. Such a climate teleconnection might have occurred through atmospheric circulation (e.g., the Westerlies). For the millennial-scale climate events during the last glacial period^1^, intensification and southward shift of the Westerlies during brief cold events in the North Atlantic have possibly caused rapid expansion of the WM, and vice versa during times of rapid warming in the North Atlantic^13,36^. The rapid climate changes in the North Atlantic might similarly influenced the East-Asian monsoon through the Westerlies during MIS 19.

## Discussion

The WM intensification occurred without anti-phase SM variation, spanning from 783‒778 ka in both Lingtai and Xifeng (Fig. 4g,h). The WM intensification in Xifeng was interrupted by the brief WM weakening event correlated with the somewhat broad SM intensification “x4” (Fig. 2b). In contrast, the intensification in Lingtai was least affected by the interruption that was quite short compared with that in Xifeng. The synchronous WM intensification would be caused by strengthening of the Siberian High, which is not ascribed to the winter insolation over Siberia^22^ (Fig. 4k). The WM intensification interval correlates with the cooling event in Osaka Bay^6,37^, ascribable to the major decrease in the earth’s magnetic dipole field to <25% of the present strength from 783 to 776 ka (Fig. 4i)^38^, from which large increases in GCR flux, by 50‒80%, can be estimated (Fig. 4j)^39^. The increased GCR flux would have induced an increase in low-cloud cover^6–8^, possibly resulting in intensification of the WM through an umbrella effect.

During the cooling event, the mean temperatures in Osaka Bay largely decreased in both the warmest and coldest months, with increases in the annual temperature ranges^37^. The mean temperature of the coldest month (MTCO) was lower than that of the warmest month (MTWA) by an average of 23.4 °C after the event, and by an additional 1.5 °C (24.9 °C in total) during the event (green in Fig. 4f). Summer precipitation decreased during the cooling event^37^. These climatic conditions, together with the WM intensification, can occur when the Siberian air mass is cooled more than the Pacific air mass in both summer and winter. In winter, the increased low-cloud cover cools the continent more than the ocean because of the differences in specific heat, causing the increased land–ocean temperature gradient to intensify the WM.

The SM did not appear to respond to the cooling event as predictably as the WM, weakening abruptly at the cooling onset at 783 ka and then recovering gradually until the sea-level peak at 780 ka. In contrast, the WM weakened for a brief interval at 783 ka and then strengthened until the end of the cooling at 778 ka with oscillations (Fig. 4c,d,g,h). Because the SM changes from S8 to S7 are consistent with the sea-level changes (Fig. 4e), the abrupt weakening of the SM at 783 ka appears to have been a response to the brief sea-level fall event possibly triggered by the cooling event.

The sea level exceeded the Osaka Bay sill between the MB and Upper Jaramillo (UJ) reversals around highstand MISs 21.3, 21.5, and 25.3, in addition to MIS 19.3 (Fig. 3)^6,40^. In the CLP, the SM peak in S9 just above the UJ correlates with MIS 25.3, just post-dating the UJ, and the L9 loess layer correlates with MISs 21–23 (Fig. 3). The latter correlation is supported by high-resolution χ and grain size data^41^. The sea-level peaks in MISs 21.3‒21.5 correlate with a relatively broad peak in the middle of the L9 layer. These correlations suggest a high mean a.r. between S7 and S8 (about 30 cm/kyr) compared with other precession-cycle intervals (<10 cm/kyr; Fig. 3c,d). The high a.r. was caused mainly by WM intensification, and partly by the long-lasting post-reversal cool, dry climate with frequent iceberg discharge in the North Atlantic^5^. The cool, dry climate suppressed pedogenesis, resulting in low χ in L8 correlated with MIS 19.2, with an interglacial sea-level elevation >−48 ± 4 m.

The accumulation rate (a.r.) of the dust was calculated between the adjacent age control points (see Method), with average intervals of 2.5 kyr for Lingtai and 2.8 kyr for Xifeng (Fig. 2h,p). The a.r. was maximal between the S8 and S7 layers at 40‒50 cm/kyr, in an interval that included the MBpf zone (Fig. 2h,p). This a.r. seems extraordinarily high compared with the 6–10 cm/kyr a.r. estimated for interglacial periods^10^ averaged over time spans >10 kyr, but it is indeed comparable with those estimated for short time spans as in this study. An a.r. of 60 cm/kyr was estimated for a time span of ca. 3 kyr, from the late Holocene paleosol layer in Weinan, the southern CLP, based on the high-resolution chronology by the 10‒20 cm interval OSL dating^29^. In addition, the a.r. values ranging from 40 to 150 cm/kyr were estimated based on the Bayesian depth-age model every 2 cm. These results indicate that a.r. depends on the time span used for the a.r. calculation, and the high a.r. of 50 cm/kyr in MIS 19 is likely even during interglacial periods. There was no significant increase in grain size fully corresponding to the increase in a.r. in Weinan. Similarly, the high a.r. interval during MIS 19 in Lingtai and Xifeng showed moderate, but no significant increases in grain size. The increases in a.r. would have been caused by extension of the dust source area, or more frequent dust storms with moderate wind speeds. Greater aridity in the source area may have been a driver, as considered for the high dust a.r. in the early Holocene^42^.

Another WM intensification event may have occurred at about 788‒784.5 ka in Lingtai (Fig. 4g). This event during the post-glacial sea-level rise consists of millennial-scale WM intensification episodes without comparable anti-phase SM episodes that were superimposed on the WM decreasing trend. It coincides with a magnetic dipole field decrease to <25% of the present strength (Fig. 4i), and thus may also reflect a GCR-induced cloud effect on the climate. The uppermost part of the L9 layer, consisting of extremely coarse dust at many sites in the CLP, including Lingtai^10,43^, correlates with the MIS 20.2 glacial maximum, and with weak (about 50% present strength) geomagnetic fields^38^. The intensive coarsening of the dust may be the result of a GCR-induced cloud effect combined with the glacial cold, dry climate.

## Method

Fine–ultrafine-grained magnetic particles neoformed by pedogenesis cause an increase in loess magnetic susceptibility (χ) and frequency dependence of magnetic susceptibility (χ_FD_)^44^. Therefore, χ and χ_FD_ are suitable proxies for SM strength (especially summer precipitation). Modern dust storms occur mainly in the spring in China, but the frequency is correlated positively with that of cold surges in the winter, a proxy for the strength of the WM^45^. Therefore, the strength of the preceding WM conditions dust storms. Hence, the grain size of dust reflects the strength of a winter north–northwesterly wind and is suitable as a WM proxy. Orbital-scale variations in χ (or χ_FD_) and the median grain size of loess show inverse correlations, both consistent with marine oxygen isotope changes^10,23^.

The acquisition of high-resolution records for the East-Asian monsoon proxies, χ, χ_FD_, and grain size, was essential for this study. We collected loess-paleosol samples from two 7.0–7.7-m-thick parallel sections in Xifeng (35.45°N, 107.49°E) and Lingtai (35.04°N, 107.39°E) in the CLP (Fig. 1). The Xifeng section had two sampling gaps about 20 cm thick because of calcareous nodule layers^15^. The average sampling intervals were 2.9 cm (Xifeng) and 2.5 cm (Lingtai) for magnetic data and 6.4 cm (Xifeng) and 4.3 cm (Lingtai) for grain size data. This dense sampling was performed to detect signals correlated with the millennial- to centennial-scale climate events reported from paleoceanic records. Detailed chronology was another critical factor for this study. Orbital tuning, a method typically applied to loess-paleosol sequences, is not sufficient for the elucidation of millennial-scale climate changes in comparison with other magnetic and climatic records on a global scale. We adopted the most detailed chronostratigraphy for MIS 19, constructed using paleoceanic records from the Northwest Pacific and North Atlantic mid-latitudes^5^. The individual methods are described in detail below.

### Magnetic and grain size analyses and data preparation

χ and χ_FD_ were measured from 2 × 2 × 2-cm^3^ specimens using a magnetic susceptibility meter SM-100 (ZH Instrument, Brno, Czech Republic). We selected 17,000 Hz as the high frequency (χ_hf_) and 500 Hz as the low frequency (χ_lf_), measured susceptibility three times for each specimen, and adopted an average. χ_lf_ was used for χ. χ_FD_ was calculated using the formula χ_FD_ = (1 – χ_hf_/χ_lf_) × 100.

For grain size analyses, organic matter and carbonate were removed from the loess and paleosol samples by adding 30% H_2_O_2_ and HCl. The treated samples were added to (NaPO_3_)_6_ as a dispersant, and their grain sizes were measured using a SALD-3000s laser diffraction particle size analyzer (SHIMADZU Corporation, Kyoto, Japan). We selected the >16-μm size fraction as a suitable index of the coarse fraction to detect faint signals of interglacial WM intensification caused by moderate-speed winds, the main target of this study. The WM intensification is insensitive to the standard indexes, >40-μm^13^ or 32-μm^23^ size fractions, which are sensitive to strong glacial-WM intensification.

For discussion, we used 3-point moving averaged χ, coarse fraction (%), median grain size, and 5-point moving averaged χ_FD_ values (Figs 2 and 4). We obtained high-pass-filtered (<50‒130 cm in wavelength) data by subtracting the 21-point moving averaged. The χ_FD_ values are more sensitive to SM than are χ values because χ_FD_ dominantly reflects components of pedogenic ultrafine grains, whereas χ reflects components of both coarse aeolian grains and pedogenic grains. Therefore, the post-reversal high-frequency changes have higher amplitudes on the χ_FD_ curves. Moreover, a weak SM interval in L8 was only <130 cm on the χ_FD_ curves, which is much narrower than the 200‒300 cm for a strong SM interval in S8 (Fig. 2c,k). However, the Xifeng χ_FD_ shows nearly flat changes around the clear peaks of SM shown by χ (Fig. 2b,c), as pedogenic components would be dominant and saturated in the χ_FD_. The cause of the saturation of χ_FD_ is not clear at present, but it may partly relate to the a.r. in Xifeng being somewhat higher than that in Lingtai in addition to precipitation.

### Climatostratigraphic correlations and age model

Loess-paleosol layers have been correlated with MISs, principally based on sequential correlations from top to bottom. The correlations until paleosol layer S6 are consistent among studies, below which they split. MIS 19 was generally correlated with S7^23,25,43^, or S8^18,30^. In high-resolution paleoceanic records, the MB boundary lies between the sea-level highstand MIS 19.3 and lowstand MIS 19.2^3,24,33^. In high-resolution loess-magnetostratigraphy records, the MB boundary lies in S8 or the S8/L8 transition, despite differences in a.r. and the degree of pedogenesis (Supplementary Fig. S2). Therefore, the correlation of S8 with MIS 19.3 is reasonable, whereas the correlation of S7 with MIS 19 requires the assumption of extraordinarily large lock-in depths of magnetization. Laboratory experiments suggest syn-depositional detrital remanent magnetization (DRM) in loess^26,27^. Even chemical remanent magnetization (CRM) carried by pedogenic ferrimagnets would be nearly syn-depositional, because the magnetic susceptibility of modern soils indicates that the majority of pedogenic ferrimagnets are formed near the surface; the magnetic susceptibility reaches mature paleosol values (>100 × 10^−8^kg^−1^) in a near-surface layer^28,29^, and shows a linear relationship with the modern precipitation^46,47^. Thus, small lock-in depths are fairly plausible, and the MB boundary provides a reliable datum level for loess-paleosol sequences.

The extremely strong WM interval with large grain sizes in the lowermost part of Lingtai (Fig. 2l,m) probably correlates with a full glacial period (MIS 20.2). Such strong coarsening is absent in the L8 layer, in which the grain size is comparable to that in the S8 layer. The median grain sizes for Xifeng and Lingtai are uniform through the S7 and S8 layers, except in the short interval around the SM minimum of L8, with a slight increase of 10–30% (Fig. 2e,m). Hence, the correlation of the L8 layer with a full glacial period is unlikely.

The short wavelength SM minima in Lingtai and Xifeng correlate with the brief cooling/low sea-level events during the MIS 19 interglacial period (Supplementary Fig. S3)^5^. Based on magnetic parameters (magnetic susceptibility [χ], anhysteritic remanent magnetization [ARM], and grain size proxy [ARM/χ]), pyrite content, and biogenic productivity, the events were originally interpreted as oxic events (or breaks of anoxia) in the North Pacific Sea bottom (Chiba Section, core TB2)^5^. They correlate well with the maxima of planktonic δ^18^O from the same core (TB2), with the low sea-level events in the diatom-based sea-level proxy curve from Osaka Bay^4^, and with the maxima of planktonic δ^18^O data from the mid-latitude North Atlantic (IODP Site 1313)^3^. Thus, we regard the oxic events as brief cooling/low sea-level events, numbered 1‒10 (Supplementary Fig. S3), and show the correlation points with small solid circles in Fig. 4b–d. The labels of 1, 3, 5, 7, 8, 9a, 9b, 10, and 10b for the events follow Hyodo *et al*.^5^, and 1a and 9′ are defined for the first time in this study. Before event 10b, two features in the North Atlantic δ^18^O curve correlate with those in the Lingtai χ_FD_ curve, shown by small solid squares (Fig. 4c).

The correlation points were dated based on the astronomical chronology for the Northwest Pacific and North Atlantic mid-latitude data^5^. The depths of Lingtai were dated by linear interpolation between the correlation points and extrapolation beyond the uppermost and lowermost correlation points with the mean a.r.s of the nearest intervals. Ages of the correlation points between Lingtai and Xifeng, shown by small solid triangles (Fig. 4c,d), were determined using the Lingtai age model. Using these correlation points together with those mentioned above, the depths of the Xifeng samples were dated in the same way as those of the Lingtai samples.

The mean a.r.s between correlation points are shown in Fig. 2h,p. They ranged from 11.8 cm/kyr to 52.4 cm/kyr for Xifeng and from 7.8 cm/kyr to 40.1 cm/kyr for Lingtai. The average time intervals for the a.r. calculations were 2.8 kyr for Xifeng and 2.5 kyr for Lingtai. Our age model shows that the upper and lower boundaries of the MBpf zone spanning 60 cm in Lingtai and 100 cm in Xifeng (Fig. 2) were consistently dated to about 777 and 779 ka, respectively. The MBpf zone must include the main MB boundary, consistent with the data for the North Atlantic, Northwest Pacific, and Osaka Bay (Fig. 4)^5^. Furthermore, the dated MBpf is consistent with the 776 ± 2 ka Ar/Ar date of the MB transitionally magnetized lavas^48^ and the mean astronomical age of 778 ka for the MB reversal^49^. The coincidence suggests small lock-in depths for the loess, as shown by observation of the latest Pleistocene geomagnetic excursions, with little time lag in well-dated loess sections^50,51^.

The successive SM features used for correlations are present at both Lingtai and Xifeng, and the age model based on the correlations provides the consistent ages for the MBpf zone between the two sites. These results support the loess-paleosol sequences analyzed herein have no hiatus.

## Supplementary information

Supplementary Information
