# Supplementary material for: Intensified East Asian winter monsoon during the last geomagnetic reversal transition
Source: Sci Rep. 2019 Jun 28;9:9389. doi: 10.1038/s41598-019-45466-8 (PMC6599209; doi:10.1038/s41598-019-45466-8)
Supplement: Supplementary file 1 — Supplementary Information [file 41598_2019_45466_MOESM1_ESM.docx]

Supplementary information

Intensified East Asian winter monsoon during the last geomagnetic reversal transition

Yusuke Ueno^1^, Masayuki Hyodo^1,2★^, Tianshui Yang^3^, Shigehiro Katoh^4^

^1^Department of Planetology, Kobe University, Kobe 657-8501, Japan

^2^Research Center for Inland Seas, Kobe University, Kobe 657-8501, Japan

^3^China University of Geosciences, Beijing, China

^4^Museum of Nature and Human Activities, Hyogo, Sanda 669-1546, Japan

^★^e-mail: mhyodo@kobe-u.ac.jp


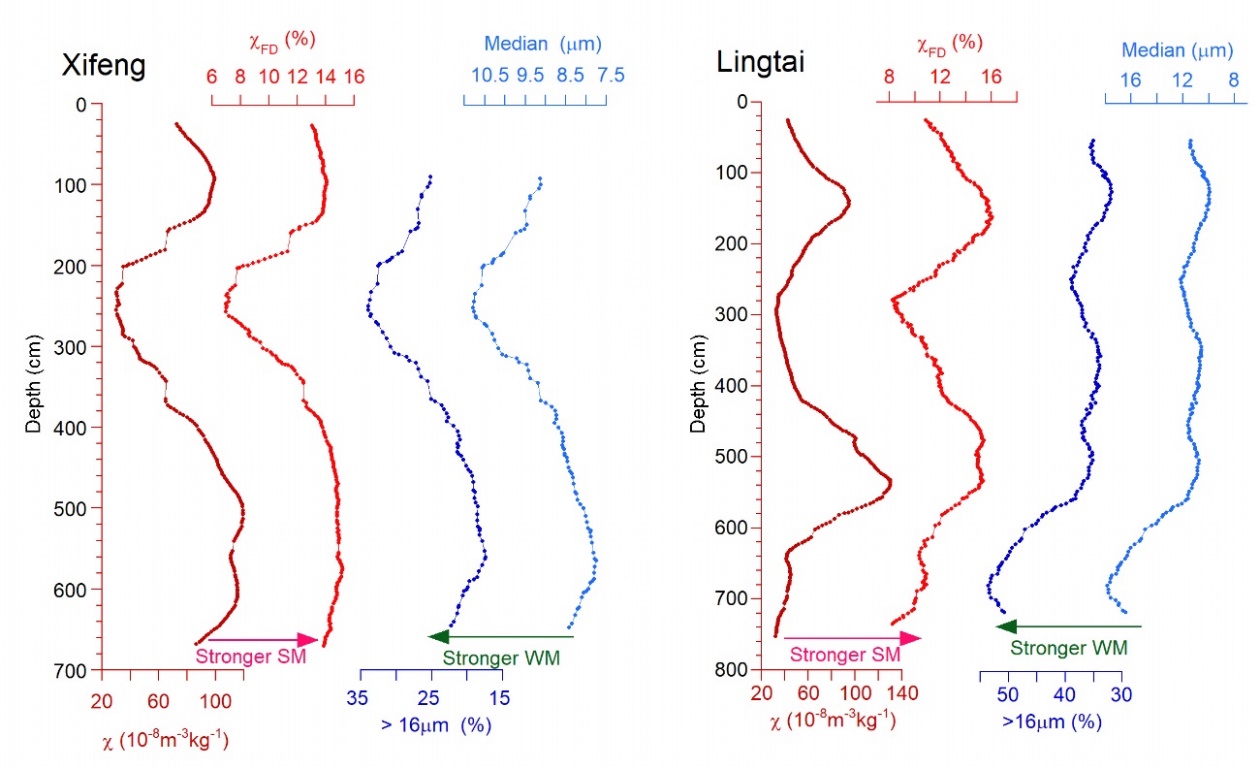


Figure S1. Down-section long-wavelength changes in the East Asian summer and winter monsoon proxies from Xifeng (left) and Lingtai (right). Plotted are the 21-point moving averaged magnetic susceptibility (χ), frequency-dependence of magnetic susceptibility (χ_FD_), content of coarse fraction (> 16μm), and median grain size data.


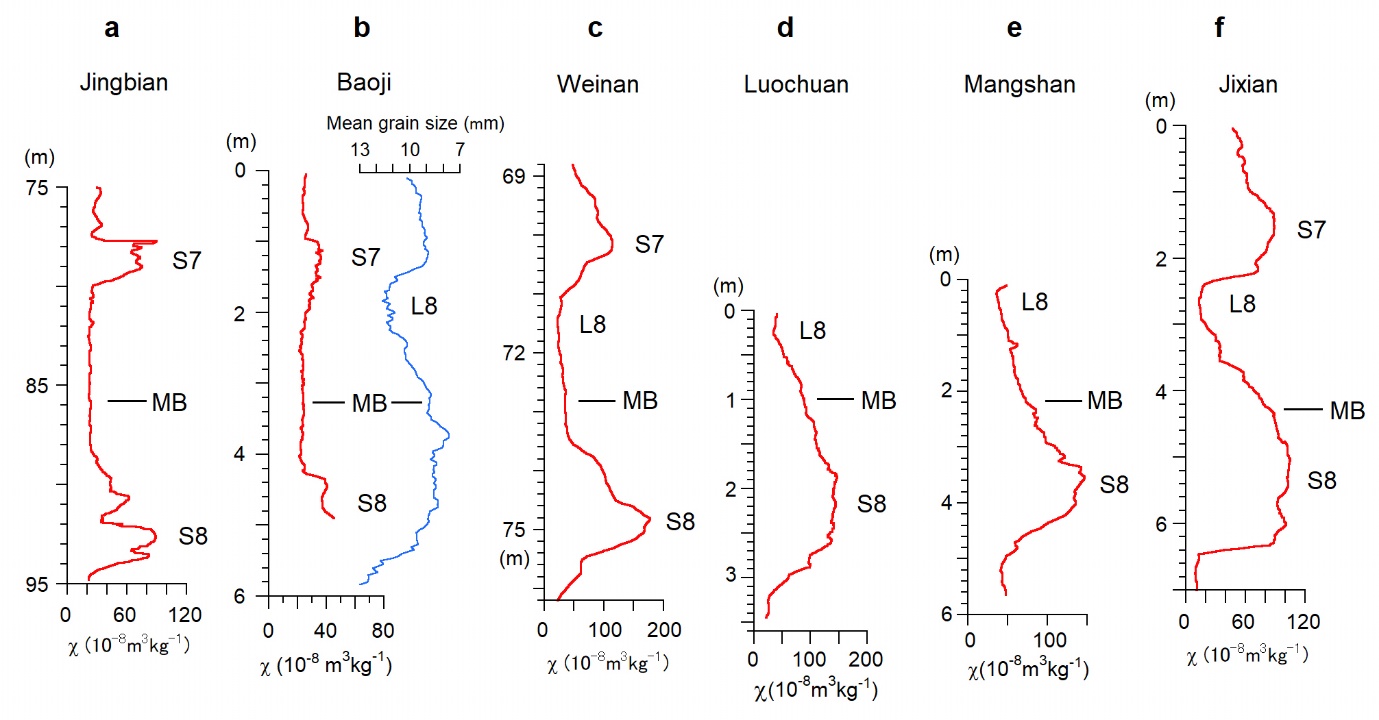


Figure S2. Magnetic susceptibility (χ) records across the Matuyama‒Brunhes magnetic polarity boundary in the loess-paleosol sequences of the Chinese Loess Plateau. **a**, Jingbian.^1^ **b,** Baoji.^2^ **c,** Weinan.^3^ **d,** Luochuan.^4^ **e,** Mangshan.^5^ **f,** Jixian.^6^ The mean grain size data from Baoji^7^ are plotted together with χ in **b**. MB shows the mid-level of the Matuyama‒Brunhes transition zone.


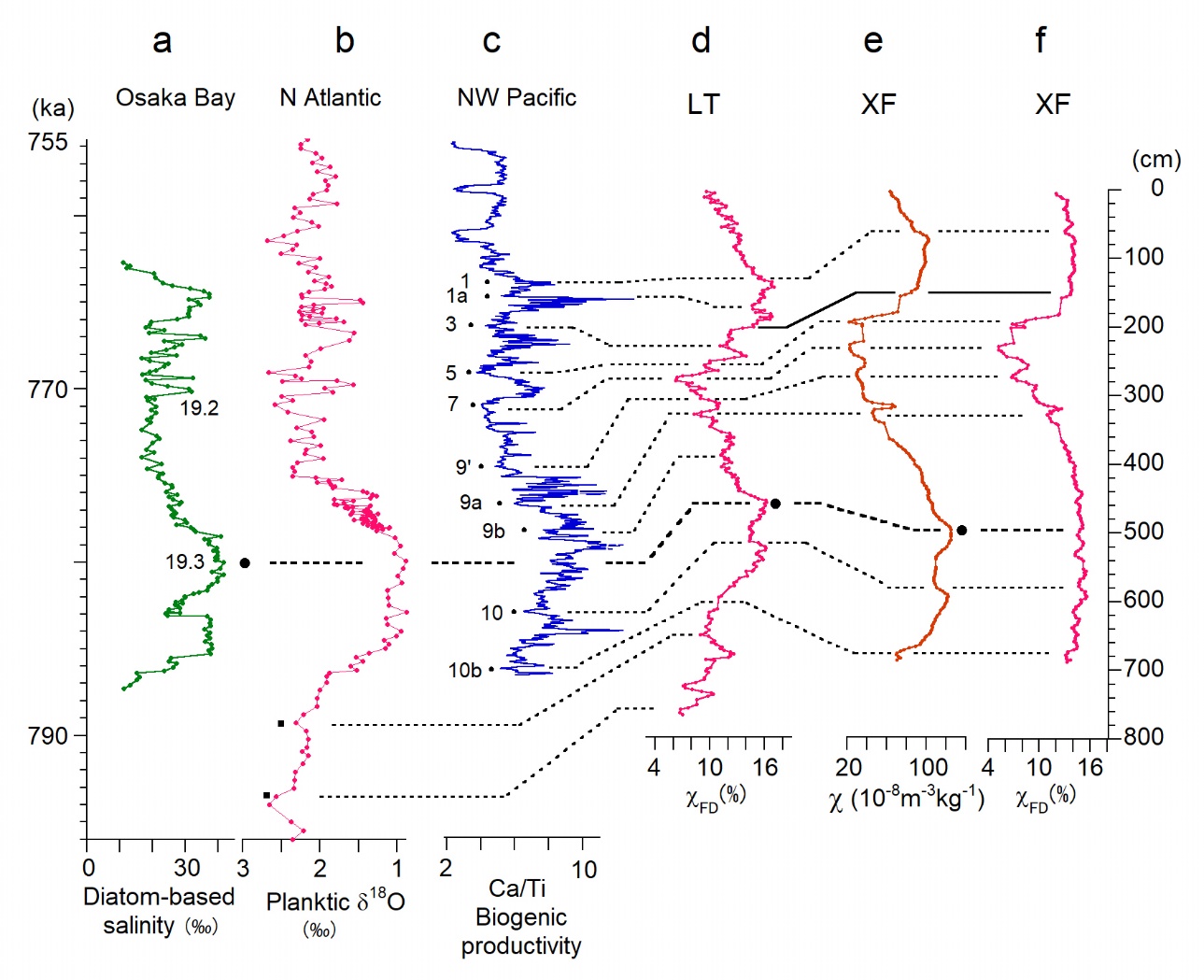


Figure S3. Climatostratigraphic correlation of the summer monsoon proxy curves from Lingtai and Xifeng with paleoceanic records. **a**, Diatom sea-level proxy from Osaka Bay.^8^ **b**, Planktonic δ^18^O from the North Atlantic mid-latitude (IODP Site U13113)^9^ plotted to the age model.^10^ **c**, Biogenic productivity proxy from the Northwest Pacific (Chiba Section core TB2).^10^ **d**, Frequency-dependence of magnetic susceptibility (χ_FD_) from Lingtai. **e**, The magnetic susceptibility (χ) from Xifeng. **f**, χ_FD_ from Xifeng. The summer monsoon maxima are correlated with the highest sea-level highstand MIS 19.3 and oxic/low sea-level/cooling events,^10^ as connected by the dotted lines. The solid line shows the correlation between the Lingtai and Xifeng data. In **c**, the labels 1 to 10b of the correlation points indicated by dots follow Hyodo et al.,^10^ except 1a and 9’ defined in this study.

Reference

1 Guo, B. *et al.* A short reverse polarity interval within the Jaramillo subchron: evidence from the Jingbian section, northern Chinese Loess Plateau. *J. Geophys. Res.* **107,** doi:10.1029/2001JB000706 (2002).

2 Yang, T.S. *et al.* Multiple rapid polarity swings during the Matuyama‒Brunhes (M‒B) transition from two high resolution loess-paleosol records. *J. Geophys. Res.* **115,** B05101. http://dx.doi.org/10.1029/2009JB006301 (2010).

3 Pan, Y.X. *et al.* Geomagnetic episodes of the last 1.2 Myr recorded in Chinese loess. *Geophys. Res. Lett.* **29,** doi:10.1029/2001GL014024 (2002).

4 Jin, C.S., Liu, Q.S. & Larrasoaña, J.C. A precursor to the Matuyama–Brunhes reversal in Chinese loess and its palaeomagnetic and stratigraphic significance. *Geophys. J. Int.* **190,** 829–842 (2012).

5 Jin, C.S. & Liu, Q.S. Revisiting the stratigraphic position of the Matuyama-Brunhes geomagnetic polarity boundary in Chinese loess. *Palaeogeog. Palaeoclimat. Palaeoecol.* **299,** 309–317 (2011).

6 Wang, X.S. The Matuyama-Brunhes polarity reversal in four Chinese loess records: high-fidelity recording of geomagnetic field behavior or a less than reliable chronostratigraphic marker? *Quat. Sci. Rev.* **101,** 61‒76 (2014).

7 Wang K. Investigation on the reasons for positional discrepancy of the Matuyama-Brunhes boundary among different loess section. *Master thesis of China University of Geosciences, Beijing*. 60 pp (2013).

8 Maegakiuchi. K. *et al.* Brief sea-level fall event and centennial to millennial sea-level variations during Marine Isotope Stage 19 in Osaka Bay, Japan. *J. Quat. Sci.* **31**, 809–822 (2016).

9 Ferretti, P., Crowhurst, S.J., Naafs, B.D.A. & Barbante, C. The Marine Isotope Stage 19 in the mid-latitude North Atlantic Ocean: astronomical signature and intra-interglacial variability. *Quat. Sci. Rev.* **108**, 95–110 (2015).

10 Hyodo, M. *et al.* Millennial-scale northern Hemisphere Atlantic-Pacific climate teleconnections in the earliest Middle Pleistocene. *Sci. Rep.* **7**, 10036 (2017).
